# A Case of Premature Delivery, with Observations

**Published:** 1803-05-01

**Authors:** 

**Affiliations:** Blackburn, Lancashire


### *A* Case *of* premature Delivery, *with Observations; communicated by* Mr. Barlow, *of Blackburn, Lancashire.*

Ellen Pickles, who resides in the village of Rishton, about three miles from Blackburn, is a woman of low stature, and when young was afflicted with rickets; she has had three children, two of the first I was constrained to extract by means of the crotchet,* owing to a distortion of the pelvis; and the life of the last, which is the subject of the following narrative, was fortunately preserved by exciting premature labour. For the purpose of effecting this mechanical event, I visited her on the 15th of January last, and requested she would dispatch a messenger for me whenever the pains of labour commenced. On the

* About the time I was employed in ascertaining the advantages derivable from exciting premature labour, together with the moral rectitude of the practice.

evening of the 16th I was sent for; and on examination per vaginam, perceived the os uteri was dilated to the circumference of a crown; the pains were strong, and had been increasing in force and frequency the greatest part of the day; the head of the fetus was movable by the pressure of the finger against the presenting cranium, consequently was not then become engaged in the pelvis. In about two hours I repeated my examination, and found the os uteri had completed its full dilatation; the head of the child was advanced some distance in the superior aperture of the pelvis, when it remained stationary for the space of two more hours, though the uterine action was very considerable. I now began to fear the child would suffer, unless some mechanical assistance could be afforded. Thus situated, I was induced to apply the lever, which in a few minutes effected the exit of the child with tolerable ease and perfect safety. The period fixed upon for exciting premature labour in this woman was as near that of the eighth month of utero gestation* as could possibly be ascertained. The child, when born, appeared lively, yet presented evident marks of immaturity, and is at this time alive and healthy; the mother's recovery was interrupted in a slight degree by a swelling of one of the lower extremities.

I have taken repeated and very accurate admeasurements of this female's pelvis, by means of the introduction of the hand, and invariably found the superior aperture from pubis to sacrum, to measure about two inches and a half, and on both sides the contraction is also perceptible, and the inferior aperture is also rendered somewhat less than natural by the projection of the os coccyx.

In order to establish the circumscribed limits wherein premature delivery is justifiable, I have in a preceding Number of the Medical and Physical Journal, dispassionately examined the comparative advantages and disadvantages of this species of birth; it may therefore appear unnecessary to expatiate further on the subject; nevertheless, so far as a single case can illustrate the point in discussion, the one above related, together with the following observations, will probably by most Accoucheurs be thought worthy a perusal. It is in this intermediate degree of distortion

† I am well aware of the difficulty of ascertaining the exact time of impregnation in the human species; but the date of the event appears more clearly established in this instance than some others, as the woman's husband had returned from the military service about the time fixed upon for the commencement of her gestation.

tion of the pelvis, requiring on the one hand the successful use of the forceps or lever, and on the other of being compelled to the dreadful alternative of terminating the delivery by Embryulcia, that I am anxious to fix its true limits; for if we exceed the bounds prescribed by either of these modes of instrumental delivery, we shall establish an abuse of the operation in question, and render our efforts of no avail to society.

The extreme shades of distortion of the pelvis are circumstances which most Accoucheurs acknowledge to occasionally exist, consequently it becomes essentially necessary to estimate the relative dimensions between the bulk of the fetal head, and the apertures of the pelvis through which it has to pass, before we have recourse to this operation. The diameter of the female pelvis (except in cases of Malacosteon) is little or none* augmented during the progress of labour, therefore our hopes must rest on the degree of compressibility which the fetal cranium is capable of sustaining; and this is so variable, that scarcely two fetal heads will be found to yield with the same facility to an equal degree of expulsive power, when passing through the pelvic canal; yet the fetal cranium will sometimes become moulded in a wonderful manner to the apertures of the pelvis; and in some instances of considerable distortion, I have observed after birth an evident depression of the cranium without manifest injury, which had been caused by the projecting angle of distortion, opposed against the head of the fetus during labour; but the depression alone, except in very long and tedious labours, will seldom become injurious; for Nature has so wisely endowed the fetal cranium with flexibility, that it will sustain considerable pressure without any palpable marks of mischief apparent on either the brain or cranium; perhaps this important organ is comparatively less affected by injury in early infancy, and before it has acquired the habit of associating the different impressions of sensation, than at a more advanced period of existence; nevertheless, if the scale of

* The opinion of the spontaneous separation of the bones of the pelvis during labour is now generally exploded; and for the satisfaction of those who wish to see the influence which this doctrine occasioned, together with the evil tendency it had on the obstetric art, may consult the works of Hippocrates, Galen, Vesalius, Harvey, Ambrose Parey, Mauriceau, Denman, and Baudelocque. Severinus Pincus, a French surgeon, who lived about the middle of the fifteenth century, collected the opinions of all the authors anterior to his time, and formed a small Treatise on the subject. This, in all probability, gave rise to the Sigaultian operation.

of difference between the diameter of the pelvis and the fetus be very considerable, insomuch that the existing disproportion amounts to three-quarters of an inch, it is highly probable that not only a depression of the cranium of the fetus may take place, but a detachment of the pericranium and dura-mater; hence internal extravasation, and consequent derangement of structure will ensue, and the functions of the brain be destroyed. I presume, therefore, it will be generally admitted, that the head of a mature fetus cannot possibly pass uninjured, through a pelvis where the antero-posterior diameter of the superior aperture, taken from pubis to sacrum, is limited to only two inches and a half; and if the dimensions be even two inches and three-quarters, considerable difficulty and danger will consequently still exist, though in a less degree; for in this state of deformity, it not unfrequently happens, that manual assistance becomes necessary; and, though dexterously accomplished, by either the forceps or lever, the fetus sometimes falls a victim thereto. It may, nevertheless, be contended by some practitioners, that in cases of distortion, as above specified, that some advantages may be obtained by reversing the fetus, and attempting delivery by the feet; but the insufficiency of effecting the exit of the fetus by this manœuvre, will very seldom authorise such attempts, as many shocking instances are recorded by authors, where either the pelvis was distorted, or some imprudent steps of the Accoucheur has eventually proved to be the cause of the loss of the fetus by this mode of delivery; such, for example, are those related by Smellie, Giffard, Dease, Nihell, Portal, La Motte, Perfect, and Ambrose Parey, where the head of the fetus was separated from the body and left in the uterus or cavity of the pelvis. Fielding Oulde gives an example well worth attention, of a woman whose pelvis was much distorted, in which case he turned the fetus with a view of facilitating delivery; unfortunately, however, he adds, "After many vain endeavours, I was at last obliged to separate the body from it (the head) by turning it round, whereby the neck was dislocated." Though the extent of distortion in this case is not precisely mentioned by the author, yet it must have been considerable; and had he obtained a competent knowledge of the diameter of the pelvis, together with the relation it bore to the fetal head, prior to turning the child, in all probability he would have saved himself some unnecessary fatigue, and, doubtless, the woman much pain, by perforating the cranium, and delivering by the crotchet.

As

As the death of the fetus, even in a well-formed pelvis, when extracted by the feet, is sometimes unavoidably the case, how much more have we to dread this event, when the head is too voluminous to traverse through the contracted apertures, and where the extent of distortion reduces the superior strait to the space of two inches and three quarters? Is it not more probable that delivery may be accomplished with greater facility and safety without turning, by the assistance of either the forceps or lever? And even these instruments, like every other obstetric invention, become subordinate, and can only be used with prudence under certain restrictions of deformity or condition; and when the pelvis measures only two inches and a half in the superior strait, it would be extremely hazardous to attempt to turn the fetus, and in most cases utterly impossible to accomplish delivery without lessening the head; and when the small diameter of the superior strait measures two inches and three-quarters, the head of the fetus may, under certain favourable conditions of structure, occasionally become moulded to the canal, and pass uninjured; yet I am disposed to conclude, that were a just parallel to be drawn of the fatality attendant on the fetus, in this last stated degree of distortion of the pelvis, I fear the balance would preponderate in the destructive scale; and granting this inference to be just, does it not appear from these data, that premature delivery offers a more favourable resource, under these circumstances, for the preservation of the fetus, than any other expedient yet had recourse to?

Manual assistance sometimes becomes necessary, even where the capacity of the pelvis bears a favourable relation to the head of the fetus. This contingent event may be caused by the head becoming propelled into an improper axis of the pelvis; and notwithstanding the expulsive efforts of the uterus, though continued for several days, would alone be insufficient to effectuate delivery; and in some rare instances, where the head has become thus locked in the passage, the crotchet will be requisite, when the succeeding labour may terminate without instrumental aid, with perfect safety to both mother and fetus. Hence it would appear manifestly absurd for the Accoucheur to conclude, that in every case where the crotchet has been used, that a similar obstacle may obtrude, and render the same resource necessary in every subsequent instance.— This circumstance undoubtedly demands the strictest attention from the Accoucheur, lest the life of the fetus, through through a mistaken zeal of acquiescence with prior practice be attended with palpable danger.

A case not very dissimilar to some above alluded to, occurred in this neighbourhood (Ramsgreave) a few years ago; the woman had been repeatedly delivered by the crotchet, by different practitioners, and her pelvis, it is said, was so much distorted on the left side* from exostoses, as not to admit any material part of the child's head to pass through it. In one instance the practitioner was induced to turn the child; but this expedient was attended with the usual fatality of her former labours, and the child became mutilated in the attempt, by the powerful exertion of the Accoucheur, and the head severed from the body; fortunately, however, the head was afterwards extracted, and the woman recovered. At the full period of her last state of utero gestation, it was my lot to attend this unfortunate woman. On my arrival I found the os uteri completely dilated, and in less than fifteen minutes a mature living child was expelled, without manual assistance. This fortunate event surprised me no little, and this more particularly on the introduction of the hand for the purpose of ascertaining the cause of this unexpected phenomena; for on the most accurate examination, I could not trace any lines of distortion to exist in any of the apertures of this female's pelvis.

This case, therefore, furnishes a striking example of the absolute necessity of ascertaining the dimensions of the different apertures of the pelvis, in every instance, prior to delivery. I trust, from what I have advanced in the preceding pages on this important subject, that the propriety of exciting premature delivery will appear in a great measure established, and induce practitioners to adopt this species of operation under the restriction of deformity of the pelvis, which I have pointed out as most conducive for the preservation of both mother and fetus.

*April* 8, 1803.

---

* For a detailed account of one of this woman's labours, see Dr. Hull's Observations on the Cæsarean Operation, Part II. p. 426—7.

